# Alkaline Phosphatase is an Independent Risk Factor for Periprosthetic Fractures in Total Joint Arthroplasty

**DOI:** 10.5435/JAAOSGlobal-D-22-00129

**Published:** 2023-02-10

**Authors:** Brandon E. Lung, Matthew Kim, Maddison McLellan, Kylie Callan, Edward D. Wang, William McMaster, Steven Yang, David H. So

**Affiliations:** From the UC Irvine Department of Orthopaedic Surgery, Orange, CA (Dr. Lung, McLellan, Callan, Dr. McMaster, Dr. Yang, and Dr. So) and the Stony Brook Department of Orthopaedic Surgery, Stony Brook, NY (Kim, and Dr. Wang).

## Abstract

**Background:**

Serum alkaline phosphatase (ALP) is a biomarker for chronic low-grade inflammation along with hepatobiliary and bone disorders. High abnormal ALP levels in blood have been associated with metabolic bone disease and high bone turnover.

**Methods:**

All primary total hip and knee arthroplasties from 2005 to 2019 were queried from the National Surgical Quality Improvement Program database. Patients with available serum ALP levels were included and stratified to low (<44 IU/L), normal (44 to 147 IU/L), and high (>147 IU/L). A risk-adjusted multivariate logistic regression was used to analyze ALP as an independent risk factor of complications.

**Results:**

The analysis included 324,592 patients, consisting of 11,427 low ALP, 305,977 normal ALP, and 7,188 high preoperative ALP level patients undergoing total joint arthroplasty. Adjusted multivariate logistic regression analysis showed high ALP level patients had an overall increased risk of readmission within 30 days of surgery compared with the control group (odds ratio [OR], 1.69; *P* < 0.01). High ALP patients also had an increased risk of postoperative periprosthetic fracture (OR, 1.6), postoperative wound infection (OR, 1.81), pneumonia (OR, 2.24), renal insufficiency (OR, 2.39), cerebrovascular disease (OR, 2.2), postoperative bleeding requiring transfusion (OR, 1.83), sepsis (OR, 2.35), length of stay > 2 days (OR, 1.47), *Clostridium difficile* infection (OR, 2.07), and discharge to a rehab facility (OR, 1.41) (all *P* < 0.05). A low ALP level was also associated with increased postoperative bleeding transfusion risk (OR, 1.12; *P* < 0.01) and developing a deep vein thrombosis (OR, 1.25; *P* = 0.03).

**Conclusion:**

Abnormal serum ALP levels in patients undergoing primary total joint arthroplasty are associated with increased postoperative periprosthetic fracture risk and medical complications requiring increased length of stay and discharge to a rehabilitation facility.

With the increase in frequency of total joint arthroplasty (TJA) in the growing elderly population, it is important to medically optimize patients before elective surgery to reduce complications, decrease length of stay (LOS), and improve outcomes. Serum alkaline phosphatase (ALP) is a comprehensive metabolic panel (CMP) laboratory value that has recently been studied as a biomarker for chronic low-grade inflammation along with hepatobiliary and bone metabolic disorders.^1^ Although previous orthopaedic research has focused on ALP for the monitoring of fracture healing and oncologic disorders, the utility of ALP as a marker of current bone mineral density, proinflammatory state, and vascular health has proven useful in stratifying patients who may be at risk of inpatient complications and fracture propagation.^1,2^ As healthcare economics shifts to outcomes-based bundled payment models, providers must understand the evolving dynamics of risk stratification for patients undergoing TJA.

A high serum ALP has been previously associated with increased fracture risk through high bone turnover and underlying decreased bone mineral density.^3^ Serum ALP may be an important predictive variable for increased risk of sustaining a periprosthetic fracture after TJA because of underlying abnormal bone metabolism. Treatment of periprosthetic fractures is complicated and typically has high mortality rates. Preoperative serum ALP may help stratify elderly patients who may need special cementation or implant-specific techniques.^4^ Moreover, emerging evidence suggests serum ALP levels serve as inflammatory markers associated with not only cardiometabolic diseases but also low-grade inflammation and overall immune function.^5,6^ In fact, serum ALP levels have been positively associated with infectious markers such as C‐reactive protein (CRP) levels and leukocyte counts, and abnormal levels may have important implications in the prevention of postoperative joint infections.^7^

Within TJA, the Centers for Medicare and Medicaid Services have focused on 30-day readmissions and complication rates as a quality indicator for value-based care. In this study, we use a large national database to determine the correlation between abnormal serum ALP levels and postoperative outcomes to inform clinical care and decision making. Although obtaining serum ALP through preoperative routine CMP is not widely adopted, this study aims to correlate abnormal ALP levels as an independent predictor for periprosthetic fractures, medical complications, and increased LOS. We predict that high serum ALP levels are associated with increased risk of periprosthetic fractures, postoperative infections, discharge to a rehabilitation facility, and longer inpatient hospital stay.

## Methods

All primary TJAs from 2005 to 2019 were queried from the National Surgical Quality Improvement Program (NSQIP) database. The database was deidentified and exempt from approval by the institution's institutional review board. Patient data were obtained from over 600 hospitals in the United States by certified healthcare workers through direct interviews, outpatient visits, and review of postoperative clinical notes.^8^ It has been reported that the inter-reliability disagreement rate is estimated to be less than 2%.^9^ Furthermore, the database is strictly audited on a regular basis to ensure its accuracy.^10^ The NSQIP database has been used for many other research studies relating to general orthopaedics,^11,12^ including joint arthroplasty.^13,14^

Current Procedural Terminology codes 27130 and 27447 were used to identify 664,599 patients who underwent TJA from 2005 to 2019. A total of 324,592 patients with available serum ALP levels were included and stratified to low (<44 IU/L), normal (44 to 147 IU/L), and high (>147 IU/L).

All statistical analyses for this study were conducted using SPSS software version 26.0 (IBM). Bivariate analysis was conducted to compare patient demographic characteristics, preoperative comorbidities, and procedural characteristics between different cohorts. A multivariate logistic regression analysis, which was adjusted for all notably associated variables such as patient demographics and preoperative comorbidities, was conducted to investigate the association between preoperative serum ALP levels and postoperative complications. Calculated odds ratios (ORs) were reported in relation to 95% confidence intervals. The level of statistical significance was set at *P* < 0.05.

## Results

Our study included a total of 324,592 patients who underwent TJA from 2005 to 2019. Of these patients, 11,427 patients (3.5%) were in the low ALP cohort; 305,977 patients (94.3%) were in the normal ALP cohort; and 7,188 patients (2.2%) were in the high ALP cohort (Table 1).

**Table 1:** Alkaline Phosphatase Cohorts

Serum Preoperative ALP	N = 324,592
Low ALP	11,427
Normal ALP	305,977
High ALP	7,188

ALP = alkaline phosphatase

In comparison with the normal ALP cohort, both low and high ALP cohorts had a greater proportion of patients with the following comorbidities: insulin-dependent diabetes, usage of hypertension medication, disseminated cancer, usage of corticosteroids, bleeding disorder, and history of transfusion. In addition, compared with the low ALP cohort, the high ALP cohort had a higher proportion of patients with the following comorbidities: insulin-dependent diabetes, dyspnea at rest and at moderate exertion, partially and totally dependent functional status, American Society of Anesthesiologists classification of three or higher, history of severe chronic obstructive pulmonary disease, ascites congestive heart failure, and usage of hypertension medication and corticosteroids as well as history of weight loss, bleeding disorder, transfusion, sepsis, and disseminated cancer (Table 2).

**Table 2:** Comparison of Patient Demographics, Medical Comorbidities, and Operative Characteristics Between Alkaline Phosphatase Cohorts

Outcome	Normal ALP	Low ALP	High ALP	*P*
Overall number	305,977	11,427	7,188	
Age (yr)	66.1 ± 10.2	66.7 ± 10.7	68.5 ± 10.5	**0.002**
BMI (kg/m^2^)	32.1 ± 6.79	30.14 ± 6.35	29.72 ± 6.67	**<0.001**

	n (%)	n (%)	n (%)	
Female	181,892 (59.4)	33,134 (62.4)	33,137 (76.4)	**<0.001**
Smoker	31,475 (10.3)	5,468 (10.3)	3,705 (8.5)	**<0.001**
Race				0.081
Black or African American	26,686 (8.7)	687 (6.0)	911 (12.6)	
Asian	4,961 (1.6)	300 (2.6)	69 (0.6)	
White	252,695 (82.6)	9,640 (84.4)	5,556 (77.3)	
American Indian or Alaskan Native	1,951 (0.7)	37 (0.4)	77 (1.1)	
Native Hawaiian or Pacific Islander	761 (0.2)	31 (0.3)	14 (0.2)	
Unknown	18,923 (6.1)	732 (6.4)	561 (8.0)	
Anesthesia				0.109
Other or none	47,265 (15.5)	1,769 (15.5)	907 (12.6)	
General	160,466 (52.4)	5,879 (51.4)	4,336 (60.3)	
Spinal	98,246 (32.1)	3,779 (33.1)	1,945 (27.1)	
Diabetes				**0.030**
Insulin-dependent	12,415 (4.1)	519 (4.5)	642 (8.9)	
Nondiabetic	254,704 (83.2)	8,927 (78.1)	5,727 (79.7)	
Non–insulin-dependent	38,858 (12.7)	1,981 (17.4)	819 (11.4)	
Dyspnea				0.003
At rest	638 (0.2)	23 (0.2)	42 (0.6)	
Moderate exertion	17,359 (5.7)	566 (5.0)	624 (8.7)	
None	287,980 (94.1)	10,838 (94.8)	6,522 (90.7)	
Functional status				0.282
Independent	299,520 (97.9)	11,212 (98.1)	6,801 (94.6)	
Partially dependent	4,931 (1.6)	160 (1.4)	317 (4.5)	
Totally dependent	239 (0.1)	8 (0.1)	23 (0.3)	
Unknown	1,287 (0.4)	47 (0.4)	47 (0.7)	
ASA classification				**0.012**
1 or 2	152,856 (50.0)	5,976 (52.3)	2,343 (32.6)	
3, 4, or 5	152,908 (50.0)	5,445 (47.6)	4,835 (67.3)	
None assigned	213 (0.1)	6 (0.1)	10 (0.1)	
History of severe COPD	12,547 (4.1)	395 (3.5)	530 (7.4)	**0.002**
Ascites	62 (0.0)	0 (0.0)	29 (0.4)	**0.004**
History of CHF	1,196 (0.4)	45 (0.4)	92 (1.2)	0.523
Use of hypertension medication	195,481 (63.9)	7,396 (64.7)	4,722 (65.7)	0.203
Disseminated cancer	907 (0.3)	47 (0.4)	158 (2.2)	0.083
Use of corticosteroids	12,964 (4.2)	630 (5.5)	522 (7.3)	**<0.001**
Weight loss	497 (0.2)	24 (0.2)	49 (0.7)	0.652
Bleeding disorder	7,565 (2.5)	312 (2.7)	345 (4.8)	0.245
Transfusion	349 (0.1)	20 (0.2)	46 (0.6)	0.181
Previous sepsis	1,188 (0.4)	38 (0.3)	97 (1.3)	**0.004**

ALP = alkaline phosphatase, ASA = American Society of Anesthesiologists, BMI = body mass index, CHF, congestive heart failure, COPD = chronic obstructive pulmonary disease

Statistically significant values (*P* < 0.05) are in bold.

An increased rate of overall complication was observed for both low ALP (9.2%) and high ALP (16.2%) compared with normal ALP (8.8%). A similar pattern was seen for bleeding transfusion (6.1%—low ALP, 11.6%—high ALP, 5.6%—normal ALP) and *Clostridium difficile* infection (0.2%, 0.2%, and 0.1%, respectively). The high ALP cohort had a higher rate of complications compared with the low ALP cohort in all complications analyzed in this study: overall complication (16.2% versus 9.2%), surgical site infection (2.2% versus 0.8%), pneumonia (1.0% versus 0.3%), renal insufficiency (0.3% versus 0.1%), renal failure (0.3% versus 0.1%), stroke/cerebrovascular accident (0.2% versus 0.1%), bleeding transfusion (11.6% versus 6.1%), sepsis (0.7% versus 0.2%), septic shock (0.3% versus 0.1%), nonhome discharge (33.8% versus 23.8%), periprosthetic fracture (0.9% versus 0.3%), readmission/revision surgery (7.4% versus 3.6%), and increased LOS of 2 or more days (84.0% versus 75.3%) (Table 3).

**Table 3:** Comparison of Complication Rates After Total Hip Arthroplasty Between Alkaline Phosphatase Cohorts

Complication	Normal ALP	Low ALP	High ALP	*P*
Overall complication	26,979 (8.8%)	1,056 (9.2%)	1,164 (16.2%)	**<0.001**
Surgical site infection	3,006 (1.0%)	89 (0.8%)	155 (2.2%)	**0.014**
Pneumonia	1,080 (0.4%)	35 (0.3%)	74 (1.0%)	**0.001**
Pulmonary embolism	1,311 (0.4%)	48 (0.3%)	19 (1.0%)	0.116
Renal insufficiency	341 (0.1%)	11 (0.1%)	24 (0.3%)	**<0.001**
Renal failure	153 (0.1%)	7 (0.1%)	23 (0.3%)	**<0.001**
UTI	2,689 (0.9%)	102 (0.9%)	78 (1.1%)	0.181
Stroke/CVA	262 (0.1%)	12 (0.1%)	14 (0.2%)	**0.007**
Bleeding transfusion	17,247 (5.6%)	696 (6.1%)	834 (11.6%)	**<0.001**
DVT	2,049 (0.7%)	96 (0.8%)	54 (0.8%)	0.069
Sepsis	738 (0.2%)	22 (0.2%)	51 (0.7%)	**<0.001**
Septic shock	195 (0.1%)	13 (0.1%)	21 (0.3%)	**<0.001**
Nonhome discharge	75,391 (24.6%)	2,714 (23.8%)	2,427 (33.8%)	**<0.001**
*Clostridium difficile* infection	194 (0.1%)	14 (0.2%)	11 (0.2%)	**<0.001**
Periprosthetic fracture	1,569 (0.5%)	36 (0.3%)	66 (0.9%)	**<0.001**
Readmission/revision surgery	11,911 (3.9%)	412 (3.6%)	530 (7.4%)	**0.001**
Increased LOS (2 or more d)	231,319 (75.6%)	8,601 (75.3%)	6,034 (84.0%)	**0.001**

ALP = alkaline phosphatase, CVA = cerebrovascular accident, DVT = deep vein thrombosis, LOS = length of stay, UTI, urinary tract infection

Statistically significant values (*P* < 0.05) are in bold.

After controlling for all associated patient demographics and comorbidities, adjusted multivariate logistic regression analysis showed that the high ALP cohort had a greater risk of developing the following complications compared with the normal ALP cohort: overall complication (OR, 1.7; *P* < 0.01), postoperative wound infection (OR, 1.8; *P* < 0.01), pneumonia (OR, 2.2; *P* < 0.01), renal insufficiency (OR, 2.4; *P* < 0.01), renal failure (OR, 4.6; *P* < 0.01), cerebrovascular disease (OR, 2.2; *P* < 0.01), postoperative anemia requiring transfusion (OR, 1.8; *P* < 0.01), sepsis (OR, 2.4; *P* < 0.01), septic shock (OR, 3.3; *P* < 0.01), discharge to a rehab facility (OR, 1.4; *P* < 0.01), *C difficile* infection (OR, 2.1; *P* = 0.02), periprosthetic fracture (OR, 1.6; *P* < 0.01), readmission/revision surgery (1.7; *P* < 0.01), and LOS > 2 days (OR, 1.5; *P* < 0.01). A low ALP level was also associated with increased risk of developing the following complications compared with the normal ALP cohort: overall complication (OR, 1.1; *P* = 0.048), postoperative bleeding transfusion risk (OR, 1.12; *P* < 0.01), deep vein thrombosis (DVT) (OR, 1.25; *P* = 0.03), and *C difficile* infection (OR, 2.1; *P* = 0.01) (Table 4).

**Table 4:** Association Between Risk of Complications After Total Joint Arthroplasty and Alkaline Phosphatase Cohort Classification

Complication	Low ALP	High ALP
	Odds Ratio *P*	Odds Ratio *P*
Overall complication	1.069 **0.048**	1.684 **<0.001**
Surgical site infection	0.766 **0.016**	1.809 **<0.001**
Pneumonia	0.857 0.375	2.236 **<0.001**
Pulmonary embolism	1.016 0.914	0.609 **0.032**
Renal insufficiency	0.800 0.468	2.390 **<0.001**
Renal failure	1.088 0.829	4.603 **<0.001**
UTI	0.999 0.989	1.109 0.388
Stroke/CVA	1.166 0.605	2.200 **0.005**
Bleeding transfusion	1.122 **0.005**	1.828 **<0.001**
DVT	1.251 **0.033**	1.084 0.565
Sepsis	0.688 0.100	2.354 **<0.001**
Septic shock	1.663 0.078	3.253 **<0.001**
Nonhome discharge	0.966 0.147	1.405 **<0.001**
*Clostridium difficile* infection	2.095 **0.011**	2.073 **0.023**
Periprosthetic fracture	0.618 **0.006**	1.624 **<0.001**
Readmission/revision surgery	0.904 0.054	1.686 **<0.001**
Increased LOS (2 or more d)	1.010 **0.008**	1.470 **<0.001**

ALP = alkaline phosphatase, CVA = cerebrovascular accident, DVT = deep vein thrombosis, LOS = length of stay, UTI = urinary tract infection

Statistically significant values (*P* < 0.05) are in bold.

## Discussion

With the new emphasis on value-based healthcare bundled payments, it is important for surgeons to adequately optimize and identify at-risk patients before elective joint arthroplasty to reduce inpatient costs, facilitate quicker functional rehabilitation, and decrease LOS.^15^ ALP is a biomarker that has previously been shown to not only correlate with the inflammatory state but also represent the current bone mineral metabolism and immune function.^16^ Identifying preoperative risk factors and risk stratification with ALP may not only help reduce inpatient hospital costs but also help guide interdisciplinary surgical planning and physician-patient communication on expected postoperative outcomes and discharge destination.^17-19^ Elderly patients undergoing arthroplasty are often malnourished with low bone mineral density and are prone to underlying disabilities, fatigue, and orthostatic hypotension, further limiting their ability to return to functional independence.^20^ With the COVID-19 pandemic limiting outdoor recreation, many patients have underlying vitamin D deficiency, which has previously been shown to be closely correlated with high ALP levels.^21^ Although preoperative ALP is available when obtaining a CMP, the significance of ALP levels remains under-recognized in preoperative arthroplasty workup and should be prioritized as a sensitive marker for predicting postoperative complications.^22^

While previous studies have highlighted the role of ALP levels in monitoring fracture healing, new literature suggests ALP may be useful as a predictor of overall bone mineral density and risk of fracture propagation.^9^ In this study, our TJA patients with high ALP levels had a markedly increased risk of sustaining a periprosthetic fracture within 30 days of surgery. High serum ALP levels have been associated with fractures through high bone turnover and negatively associated with bone mineral density.^10^ In fact, a low ALP level in our patients was protective of sustaining a periprosthetic fracture, which suggests abnormally high ALP levels may reflect an underlying abnormality in bone quality and density that may not be able to withstand stresses from prosthetic impaction, reaming, broaching, and early weight bearing.^23^ Perhaps owing to poor bone healing and underlying malnourishment, our high ALP level patients had an increased risk of discharge to a skilled nursing facility, longer LOS, and 30-day readmission. The extended stay seen in patients with high ALP levels is important to consider from a billing, hospital bed space, and hospital quality metrics perspective. Understanding the expected discharge disposition planning is important in our aging population with multiple comorbidities and is the initial step in being able to achieve interdisciplinary cost-effectiveness and better use of limited resources.

Not only has serum ALP been shown to be a marker for bone disorders but also has recently been discovered to be a biochemical marker of cardiometabolic disease and low-grade inflammation.^8^ In this study, patients with high preoperative ALP levels had markedly increased risk of postoperative infectious complications, including surgical site infection (SSI), pneumonia, sepsis, and *C difficile* infection. Emerging evidence indicates that serum ALP is considered an inflammatory mediator and positively associated with established infectious markers such as CRP levels and leukocyte counts.^14,24^ High proinflammatory states are linked to a greater risk of hospitalization, and high ALP levels are seen with dysregulation of osteoblast and osteoclast development needed for B-cell maturation and overall immune function.^25^ Although in this study low ALP levels were protective of obtaining postoperative SSIs, low ALP levels were also associated with postoperative *C difficile* infection and septic shock, which may suggest global abnormal ALP levels have an underlying effect on the proinflammatory state and immune functional integrity.

Previous associations of serum ALP and bone metabolism disorders have led researchers to support the notion of a bone-vascular axis linking vascular calcification with abnormal serum ALP and overall bone health.^26-28^ In our study, patients with abnormal ALP levels had notable increased risk of postoperative complications of a cerebrovascular accident and DVT. Greater severity of aortic and vascular calcification has been seen with high ALP levels, and in the literature, higher serum ALP levels are associated with increased all-cause and cardiovascular mortality.^29-31^ The relationship of ALP and vascular health is important to consider in our study because patients with preoperative high ALP levels had nearly double the risk of normal ALP patients in developing postoperative stroke and cerebrovascular accident. Although we predicted the rate of DVT to be higher in the patients with high ALP levels due to increased vascular smooth cell activation, we found low ALP levels to be predictive of postoperative DVT.

Overall, although it is difficult for providers to identify at-risk patients for postoperative periprosthetic fractures and complications, this study suggests the utility of obtaining a CMP before elective arthroplasty to evaluate serum ALP levels. It is important for healthcare providers to understand the implications of a high ALP level and understand that serum levels may also be reflective of patients with biliary obstruction, colon cancer, liver disease, vitamin D deficiency, and Paget disease.^21,32,33^ Abnormal ALP levels during preoperative visits may alert and help providers to suspect underlying metabolic disease and seek additional medical workup and optimization before elective arthroplasty. Understanding patient expectations and identifying comorbidities is an important part of being able to improve outcomes and prevent unexpected complications. With the increasing use of ALP as a biomarker for underlying bone mineral dysregulation, previous studies have advocated for increased vitamin D supplementation, smoking cessation, liver protective supplementation, alcohol cessation, and daily intake of vegetables such as broccoli, onions, and cabbage as ways to proactively improve ALP levels.^34-36^ However, future studies are needed to investigate whether serum ALP levels can be normalized in a realistic time line before elective arthroplasty.

Despite the large number of patients included, there are limitations to consider when using the NSQIP database, including selection bias. Although the national database is unable to differentiate bone-specific ALP from serum ALP, not all studies have confirmed the value of bone-specific ALP as a marker of bone formation and may be because of differences in study populations.^37^ In fact, there is a mixed consensus on the role of bone-specific ALP in predicting bone mineral density and risk of fractures.^38^ Although the data comprise a heterogeneous population nationwide at different ambulatory settings, the wide variety of ambulatory centers and surgeon expertise may confound recorded outcomes. Although various institutions may implement different preoperative pathways for joint arthroplasty, patients from both academic and private practice settings in rural and urban centers reflect the generalizability of our results. Furthermore, it is possible that we were not able to record all cases of postoperative periprosthetic fractures because the database is limited to short 30-day complication rates. Final limitation of this study is not considering inflammatory markers such as erythrocyte sedimentation rate and CRP, which are often linked with abnormal ALP levels because the data were not available in the NSQIP database.

## Conclusion

Overall, ALP is an important preoperative diagnostic tool to identify at-risk patients with underlying bone mineral metabolism disorder and inflammation undergoing elective joint arthroplasty. ALP is often an overlooked risk factor, and this study suggests the utility of obtaining a preoperative CMP to identify patients who may be at increased risk of sustaining postoperative periprosthetic fractures, increased LOS, and medical complications. As the landscape of healthcare economics shifts to value patient satisfaction outcomes, discharge destination, and reduced hospital costs, ALP is important to consider as part of a stratified pathway protocol for a growing elderly population with inherent medical comorbidities.
